# Chiropractic treatment including instrument-assisted manipulation for non-specific dizziness and neck pain in community-dwelling older people: a feasibility randomised sham-controlled trial

**DOI:** 10.1186/s12998-018-0183-1

**Published:** 2018-05-10

**Authors:** Julie C. Kendall, Simon D. French, Jan Hartvigsen, Michael F. Azari

**Affiliations:** 1 School of Health and Biomedical Sciences, RMIT University, PO Box 71 Bundoora, Melbourne, VIC 3083 Australia; 2 School of Rehabilitation Therapy, Queens University, Kingston, Canada; 3 Department of Chiropractic, Macquarie University, Sydney, Australia; 4 Department of Sports Science and Clinical Biomechanics, University of Southern Denmark, Odense, Denmark; 5 Nordic Institute of Chiropractic and Clinical Biomechanics, Odense, Denmark

**Keywords:** Neck pain, Dizziness, Elderly, Chiropractic, Randomised controlled trial

## Abstract

**Background:**

Dizziness in older people is a risk factor for falls. Neck pain is associated with dizziness and responds favourably to neck manipulation. However, it is unknown if chiropractic intervention including instrument-assisted manipulation of the neck in older people with neck pain can also improve dizziness.

**Methods:**

This parallel two-arm pilot trial was conducted in Melbourne, Australia over nine months (October 2015 to June 2016). Participants aged 65–85 years, with self-reported chronic neck pain and dizziness, were recruited from the general public through advertisements in local community newspapers and via Facebook. Participants were randomised using a permuted block method to one of two groups: 1) Activator II™-instrument-assisted cervical and thoracic spine manipulation plus a combination of: light massage; mobilisation; range of motion exercises; and home advice about the application of heat, or 2) Sham-Activator II™-instrument-assisted manipulation (set to zero impulse) plus gentle touch of cervical and thoracic spinal regions. Participants were blinded to group allocation. The interventions were delivered weekly for four weeks. Assessments were conducted one week pre- and post-intervention. Clinical outcomes were assessed blindly and included: dizziness (dizziness handicap inventory [DHI]); neck pain (neck disability index [NDI]); self-reported concerns of falling; mood; physical function; and treatment satisfaction. Feasibility outcomes included recruitment rates, compliance with intervention and outcome assessment, study location, success of blinding, costs and harms.

**Results:**

Out of 162 enquiries, 24 participants were screened as eligible and randomised to either the chiropractic (*n* = 13) or sham (*n* = 11) intervention group. Compliance was satisfactory with only two participants lost to follow up; thus, post-intervention data for 12 chiropractic intervention and 10 sham intervention participants were analysed. Blinding was similar between groups. Mild harms of increased spinal pain or headaches were reported by 6 participants. Costs amounted to AUD$2635 per participant. The data showed a trend favouring the chiropractic group in terms of clinically-significant improvements in both NDI and DHI scores. Sample sizes of *n* = 150 or *n* = 222 for dizziness or neck pain disability as the primary outcome measure, respectively, would be needed for a fully powered trial.

**Conclusions:**

Recruitment of participants in this setting was difficult and expensive. However, a larger trial may be feasible at a specialised dizziness clinic within a rehabilitation setting. Compliance was acceptable and the outcome measures used were well accepted and responsive.

**Trial registration:**

Australian New Zealand Clinical Trials Registry (ANZCTR) ACTRN12613000653763. Registered 13 June 2013.

Trial funding: Foundation for Chiropractic Research and Postgraduate Education (Denmark).

## Background

Dizziness and musculoskeletal pain are common in older people [1–4] and are associated with postural instability [5], fear of falling [6–8], and increased incidence of falls [9–12]. Among Australian adults, 36% of people aged over 50 years report the presence of dizziness within the last month [13]. Similarly, the prevalence of neck pain in Australian older adults has been estimated at 36% and 41% for men and women respectively [14], and in older adults, 5% of men and 8% of women report that neck pain interferes with their physical activity [2].

Dizziness is a known risk factor for falls in community-dwelling older people [11], and dizziness is not optimally managed at present. One in three older people with dizziness are prescribed medications that are known to increase the risk of falling including: anti-hypertensives; anxiolytics and antidepressants; nitrates; analgesics; and anti-vertigo medications [15]. Anti-vertigo medications in particular, are commonly prescribed for non-vestibular causes of dizziness [16]. Therefore, there is a need to develop and validate non-pharmacological treatment strategies for dizziness in this population, which in turn may reduce the need for prescription of pharmacological agents with their attendant potential side-effects [15, 17].

Neck pain may increase the risk of falls in the elderly. Sensory information from various structures including the vestibular apparatus in the inner ear, the eyes, and the proprioceptive receptors in muscles and joints, particularly of the neck, is integrated by the brain for position sense, balance and motor control [18]. In some individuals, neck pain is linked with dizziness, in a syndrome termed ‘cervicogenic dizziness’ or ‘cervical dizziness’ [19–21]. There are reports in neck pain patients of a correlation between cervical joint stiffness and hypertonicity of the upper cervical musculature and the presence of dizziness [22–24]. There are many different causes of dizziness in older people, including vestibular, cardiovascular, and psychological [25]. In addition, since cervicogenic dizziness is a diagnosis of exclusion, its exact incidence and prevalence remain unknown and a definitive diagnosis of cervicogenic dizziness is not possible in primary care settings. Therefore, this study recruited older people who reported both chronic non-specific dizziness and chronic neck pain to optimise the relevance of the study findings for primary care settings.

Spinal manipulative therapy (SMT) is widely used by chiropractors, osteopaths and physiotherapists, for musculoskeletal conditions, including neck pain [26]. SMT has been shown to reduce neck pain in adults in general [27] and in older people specifically [28]. Furthermore, there have been small studies reporting positive effects of manual therapy in improving dizziness and musculoskeletal pain in older people [29–31]. In fact, there is evidence that dizziness specifically associated with neck pain in adults may be attenuated with manual therapy, including SMT [32, 33]. However, previous studies have several important limitations including: lack of specific examination of neck pain [29–31]; lack of a control group [29, 30, 34]; use of a ‘no treatment’ control group [31], issues with appropriate outcome measures [35] and small sample sizes. SMT can be performed manually or assisted through an instrument. There is some evidence suggesting that low-force Activator™-instrument-assisted manipulation may produce effects on musculoskeletal pain that are comparable to those of manual SMT [36]. Even though reported significant harms following neck SMT in older people are rare [37], due to increased risk of osteoporosis in this population, low-force SMT techniques are recommended by recent chiropractic guidelines [38].

A feasibility or pilot study is generally recommended before a Phase III clinical trial [39]. Feasibility studies can determine the efficiency of recruitment strategies, adequacy of randomisation and blinding, appropriateness of outcome measures, and acceptability of compliance levels, as well as give some indication of the frequency and nature of harms. In this way, it is possible to ensure that the full-scale trial makes efficient use of resources, and is sufficiently powered to provide meaningful results [40].

We conducted a feasibility randomised sham-controlled trial (RCT) of a chiropractic intervention including instrument-assisted SMT in older people with chronic dizziness and concomitant chronic neck pain. The primary objective of this trial was to test the feasibility of a fully-powered RCT, based on recruitment rates, compliance with intervention and outcome assessment, study location, success of blinding, costs and harms. The secondary objective of this trial was to calculate, based on observed group differences, sample sizes for fully-powered RCTs using the dizziness handicap inventory (DHI) or neck disability index (NDI) as the primary outcome measures.

## Methods

### Study design

We conducted a parallel two-arm, randomised, sham-controlled feasibility trial. Participants were allocated to either a chiropractic intervention or sham intervention using a block randomisation procedure.

### Ethics and trial registration

The human ethics clearance was obtained from RMIT University’s human research ethics committee (HREC) (Approval number 29/13). The trial was registered in the Australian New Zealand Clinical Trials Registry (ANZCTR) (Registration number: ACTRN12613000653763).

### Participants

#### Recruitment

Participants were recruited from the northern Melbourne metropolitan region via notices in ‘The Leader’ local newspapers (covering Diamond Valley, Hume, Whittlesea, and Moreland municipal areas), flyers at local community centres surrounding the research location, flyers at RMIT university departments, and targeted online Facebook advertisements. Potential participants who responded to these recruitment methods were interviewed over the telephone to determine eligibility for study enrolment. If eligibility could not be determined via telephone alone, potential participants were invited to the university campus for further examination by a research assistant, who was a registered chiropractor.

#### Inclusion criteria

Participants included in the study were men and women aged between 65 and 85 years. Participants had to report having neck pain with concomitant dizziness (described as dizziness or unsteadiness), at least of three-months duration each. Pain and dizziness could be constant or intermittent within the previous three months.

#### Exclusion criteria

Participants were excluded if they self-reported: diagnosed vestibular pathology such as Meniere’s disease or benign paroxysmal positional vertigo; a history of cerebrovascular accident or myocardial infarct; psychiatric disease; active inflammatory spondyloarthropathies (e.g. rheumatoid arthritis, psoriatic arthritis, ankylosing spondylitis); recent spinal trauma; osteomyelitis; spinal tumours; acute myelopathy; and if they had received neck any SMT or neck massage during the previous three months. Participants were excluded if they showed signs of cognitive impairment as demonstrated by a Montreal Cognitive Assessment (MoCA) score of 20 or less [41].

### Interventions

All interventions, including the sham intervention, were delivered by either one of two registered practicing chiropractors (depending on availability) who each had at least 20 years of clinical experience. Four intervention sessions were given over four weeks, and the duration of each session was kept to 15 min in both groups.

#### Chiropractic intervention

Chiropractic care included: Activator II™ instrument-assisted manipulation plus one or more of the following: joint mobilisation; massage; range of motion neck exercises; or advice to apply heat at home. Interventions were directed to cervical and thoracic joints that displayed local tenderness and/or areas of joint stiffness in accordance with common chiropractic practice [42]. The chiropractor administered the instrument-delivered thrust following pre-tensioning of hypo-mobile cervical joints in lateral flexion without extending or rotating the neck [43]. Instrument-assisted manipulations to the thoracic spine were delivered in the prone position. Manipulation with or without mobilisation was supplemented with massage to hypertonic muscles of the cervical and thoracic spine, as determined by the clinical judgment of the practitioner, as well as advice on local application of heat at home. Massage consisted of a combination of effleurage, and ischaemic compression techniques. The intervention approach was designed to reflect actual contemporary Australian chiropractic care (unpublished data) and recent chiropractic practice guidelines [38].

#### Sham intervention

Activator II™ instrument impulses (set at zero) and gentle placement of the practitioner’s hands on the cervical and thoracic spine regions. This was a modification of a published sham procedure [44]. No massage, mobilisation, or home advice was given to the participants in the sham group.

### Outcome measures

#### Feasibility outcome measures

Feasibility of running a larger trial was determined based on recruitment rates, compliance with intervention and outcome assessment, reviewing the study location, blinding, costs and reporting of harms.

### Recruitment rate

The recruitment rate was determined by comparing the number of enquiries from each advertising method with the number of participants who were enrolled from each of those methods. Additionally, the inclusion and exclusion criteria were reviewed by examining the frequency and the reasons for exclusion during screening.

### Compliance

Participants’ compliance with the outcome assessment was examined by noting the time taken to complete the baseline questionnaires and assessments, and reviewing if any outcome measures were consistently filled out incorrectly or were incomplete. Compliance with the intervention schedule was examined by measuring drop-out rates and reasons for drop-outs.

### Study location

We determined if participants dropped out, or had difficulty finding the clinical trial centre, due to its location on the Bundoora campus of RMIT University.

### Blinding and treatment satisfaction

At the conclusion of the follow-up outcome measure assessment, participants indicated which intervention group they believed they were in (sham or chiropractic) to determine the integrity of allocation. All participants were also asked to rate their satisfaction with treatment on a five-point scale (from 1: I feel much worse to 5: I feel much better).

### Costs

The feasibility of conducting a larger, fully-powered study was assessed on the basis of the costs of: advertising, equipment, and hiring the chiropractors and research assistants to recruit and screen potential participants and administer the interventions.

### Harms

Harms were defined as adverse consequences of the intervention reported by participants. In accordance with the World Health Organisation’s [45] *Conceptual Framework for the International Classification for Patient Safety*, the degree of harm was classified as: none (no symptoms detected and no treatment required), mild (minimal or intermediate short term harm caused, and minimal or no intervention required), moderate (permanent or long-term harm caused, or intervention required), severe (major permanent or long-term harm caused, or major surgical/medical or life-saving intervention required) or death (death caused or brought forward). Harms and other reactions to interventions were documented by the treating chiropractor at each intervention session.

#### Clinical outcomes

Clinical outcomes were assessed at the baseline visit (one week pre-intervention) and follow-up visit (one week post-intervention). Dizziness, pain, quality of life, mood, and concerns of falling were assessed with self-reported questionnaires. The physical function and mobility assessments were performed one after the other, and participants were able to take breaks in between if they became tired. During all physical function tasks, the investigator stood close by to assist/steady the participant as required. These outcome measures were chosen to explore potential clinical measures that may show improvement of pain and dizziness to be utilised in a larger trial as primary (neck pain or dizziness) or secondary (quality of life, mood and physical function) outcomes.

### Sample size

As a feasibility study, this trial did not have an a priori calculated sample size. We aimed for a sample size of 40 to generate sufficient information to address the feasibility objectives, particularly the recruitment rate.

### Randomisation

Participants were randomly assigned after the baseline appointment using a permuted block randomisation protocol. The randomisation schedule was conducted by an independent statistician before recruitment using a computer generated random list of numbers. This list was used to assign random blocks of four or six participants at a time. Group allocations were concealed by placing them in opaque consecutively numbered sealed envelopes, which were opened, in order, by the treating chiropractor before the first intervention session.

### Blinding

Participants were blinded to group allocation. The researcher performing the outcome assessments was also blinded to group allocation. The chiropractors involved in performing the interventions were blinded to the results of the outcome assessments at pre- and post-intervention.

### Analysis

#### Feasibility determination

A fully powered RCT using this protocol would be determined a priori to not be feasible in our setting if:

- At least 40 participants could not be recruited within the three-month trial period.
- More than 20% of all participants could not participate for the following reasons:
  i. Primarily identifying travelling to the study location as inconvenient;
  ii. Being unable able to complete all outcome measure assessments, or unable to complete them within the allocated two hours;
  iii. Becoming lost trying to find the study location.
- More than 15% of participants in either group dropped out.
- More than 70% (20% greater than chance) of participants in either group correctly identified their allocation at post-intervention follow-up, or blinding was significantly different between groups.
- The average cost of recruitment and intervention per participant was more than $1500.
- There were any reported severe harms.

A fully-powered RCT was determined to be feasible with modifications if any of the above criteria were not satisfied but could be modified in such a way as to preserve the integrity of the study.

#### Statistical analysis

To address the primary objective of the trial, descriptive statistics were calculated and reported for all feasibility and clinical outcome measures. Pre- and post- intervention self-reported and test outcomes were calculated for each group with means and standard deviations. A chi-squared test was performed to determine if blinding was similar between groups, using IBM SPSS software (version 22). To address the secondary objective of the trial, two sample size calculations for possible larger, fully-powered trials were conducted at the conclusion of this trial with NDI or DHI as the primary outcome measure. Sample size was calculated in G*Power (version 3.1.9.2) to estimate an a priori two-tailed independent two-group mean difference using the effect size (cohen’s *d*) estimated from the NDI and DHI data based on means and standard deviations of post-intervention scores for each group. Sample size was estimated for a power set to 80%, and significance level of 0.05. Since this was a feasibility study, we did not perform any statistical analysis to determine effectiveness of the chiropractic intervention.

## Results

### Recruitment

**Fig. 1:**
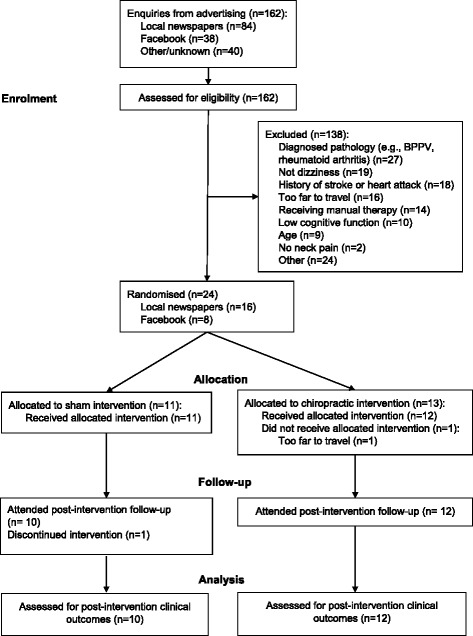
Flow of participants through the trial

A total of 24 participants were recruited from 162 telephone enquiries (Fig. 1). The recruitment period ran over nine months, from October 2015 to June 2016; six months over the planned three-month period. Most commonly, screened participants were excluded due to the presence of self-reported diagnosed vestibular and spinal pathologies (*n* = 27 [20%]), neck pain without symptoms of dizziness (*n* = 19 [14%]), history of cardiovascular incidents (*n* = 18 [13%]), recent manual therapy (*n* = 14 [10%]) and not being able to travel to the research site (*n* = 16 [12%]). Ten participants (7%), who otherwise could have participated, were excluded due to low performance on the MoCA cognitive function assessment.

Using Facebook as a recruitment method for older Australians was surprisingly successful, with 38 (23%) enquiries; however, it captured many potential participants who were unable to travel to the trial location. The trial was stopped before the target 40 participants was reached due to time and budget constraints.

### Compliance

Clinical outcome measurements took between 60 and 90 min to complete for most participants, and all participants took less than the 2 h that was allocated. Participants were offered a break if they became tired, but this proved to be unnecessary in all cases. Questionnaires were checked by the investigator who pointed out several questions that were often missed and asked the participant to complete them.

After enrolment in the trial, participant compliance with the four interventions were deemed acceptable, with only two drop-outs. One drop-out from the sham group had a spontaneous aggravation of a lower back complaint unrelated to intervention and another participant in the chiropractic intervention group did not start the intervention due to inability in making the travel commitment.

### Location

Travel to the outer-suburban university location was a barrier for 16 (12%) potential participants. Additionally, one drop-out was due to difficulties with travel. Participants often became lost on their initial visit to the campus. While temporary parking permits were provided, the participants had to pick these up from the security station. This was sometimes confusing for them.

### Blinding and overall improvement

Blinding was similar between groups, with 5 (50%) participants in the sham group and 8 (67%) participants in the chiropractic group correctly identifying which intervention they received [chi-squared = .627, *p* = 0.361 (minimum expected count 4.09)]. Both the chiropractic group and the sham group were equally satisfied with the care they received [mean (SD): chiropractic 3.58 (1.0); sham 3.6 (0.7)], indicating that the sham protocol provided sufficient patient satisfaction.

### Costs

Advertising costs totalled AUD$43,679. Minor equipment costs were AUD$395. Two registered chiropractors were employed part-time as research assistants to set up the procedures, screen participants and quantify outcome measures; the cost for this was AUD$3033. In addition to one of the investigators (MFA), another experienced registered chiropractor was employed to be available to provide the weekly interventions at a cost of AUD$10,866. Therefore, total costs amounted to AUD$57,973. This translated to AUD$2635 per participant. The costs (per participant) of recruitment and intervention were AUD$2141 and AUD$494 respectively. These costs excluded the salary of the senior author (MFA) and the PhD scholarship of the first author (JCK).

### Harms

Six (27%) participants reported harms. All harms were mild, including increased neck pain (chiropractic *n* = 2, sham *n* = 1), headache (chiropractic *n* = 1, sham *n* = 1) and mid-back pain (chiropractic *n* = 1).

### Clinical outcomes

Many clinical outcome measures were used in this study (Table 1). Participants had moderate intensity of dizziness at baseline [mean (SD)] in both the chiropractic group and the sham group (Table 2). DHI scores were also similar at baseline and improved in both groups post-intervention [chiropractic 28.33 (14.37) to 40.77 (12.48); sham 44.00 (16.97) to 36.40 (20.11)]. Similarly, NDI scores were reduced post-intervention [chiropractic 24.94 (12.87) to 19.07 (12.50); sham 24.18 (8.22) to 22.8 (6.2)]. Fifty eight percent of the chiropractic group showed a clinically-significant improvement (of at least 19%) in NDI scores compared to 30% of the sham group (Table 3). The DHI scores improved by the clinically significant amount (of at least 18%) in 67% of the chiropractic group compared to 50% of the sham group. Mood was generally low, with participants commonly reporting symptoms of depression, anxiety and stress on the DASS. Concerns of falling were high in both groups at baseline [chiropractic 26.00 (5.61); sham 29.00 (5.71)], and reduced slightly in both groups [chiropractic 24.42 (5.21); sham 26.7 (6.29)]. All participants were able to complete the physical functional tasks.

**Table 1:** Descriptions of clinical outcome measures

Clinical outcome	Outcome measure	Description	Scoring
Dizziness	Numerical rating scale (NRS11)	Participants were asked to rate their dizziness experiences ‘today’ with 0 indicating no dizziness and 10 very severe dizziness.	0–10
Dizziness	Dizziness Handicap Inventory (DHI)	DHI is a comprehensively validated measure of disability due to dizziness from a range of causes [48], and has demonstrated responsiveness to chiropractic interventions in older people [30].	0–100
Neck pain	NRS11	Participants were asked to rate their neck pain experience ‘today’ from 0 (no pain) to 10(very severe pain).	0–10
Neck pain	Neck disability index (NDI)	NDI is a 10 item questionnaire reporting pain and difficulties with everyday activities [49].	0–100
Quality of life	SF12	SF12 is a 12-point questionnaire that gives two combined scores: a physical component score (PCS) and a mental component score (MCS).	PCS: 0–50 MCS: 0–50
Mood	Disability Anxiety Stress Scale (DASS21)	DASS contains 21 questions that report depression, anxiety and stress symptoms within the past week. Each component is scored separately. Interpretation is as follows: depression normal 0–4, moderate 5–8, severe 9–12 extremely severe 13–21; anxiety normal 0–3, moderate 4–6, severe 7–9, and extremely severe 10–21; stress normal 0–6, moderate 7–11, severe 12–16, and extremely severe 17–21 [50].	Depression 0–21 Anxiety 0–21 Stress 0–21
Concerns of falling	Falls Efficacy Scale International (FES-I)	FES-I is a 16 item questionnaire measuring the level of concern of falling undertaking activities and routines [51].	16–64
Cognitive function	Montreal Cognitive Assessment (MoCA)	MoCA is a 10-min screening assessment for cognitive impairment. The domains are: attention and concentration, executive functions, memory, language, visuo-constructional skills, conceptual thinking, calculations, and orientation.	0–30
Physical function, mobility and balance	Step test	The number of times a person can repeatedly step one foot up and down on and off a standard 7.5 cm height step in 15 s [52]. Both feet were tested and a combined score was used.	Number of steps.
	Timed Up and Go (TUG)	A measure of the time taken to stand up from a standard height armchair, walk a distance of three metres, turn, return and sit back down in the chair [53].	The time taken in seconds.
	Functional reach	The distance an individual can reach forward with their dominant arm extended at horizontal, while standing. This is scored with the difference between starting reach and furthest reach in centimetres [54].	The distance in centimetres.
	Four-square step test	The time taken to step in a sequence of forward, to the left, backwards and to the right, and then reversed. Each step is performed over an obstacle to increase difficulty [55].	The time taken in seconds.

**Table 2:** Pre- and post- intervention clinical outcome measures

	Sham group				Chiropractic group			
	Pre n = 11		Post n = 10		Pre n = 13		Post n = 12	
Female (n)	5	(46%)	5	(50%)	6	(46%)	5	(42%)
Age (years)	72.55	(4.27)	72.9	(4.33)	74.23	(5.83)	73.75	(5.82)
Cognitive function (MoCA) (0–30)^a^	26.09	(1.92)	26.90	(1.60)	26.00	(2.16)	26.25	(2.67)
Dizziness (0–10) ^b^	4.00	(3.58)	3.50	(2.88)	3.85	(2.12)	2.58	(2.64)
Dizziness handicap (DHI) (0–100)^b^	44.00	(16.97)	36.40	(20.11)	40.77	(12.48)	28.33	(14.37)
Joint pain (NRS-11) (0–10)^b^	2.27	(2.33)	3.30	(2.21)	2.69	(2.02)	2.92	(2.84)
Neck pain (NRS-11) (0–10)^b^	2.82	(1.78)	3.60	(2.12)	4.38	(2.36)	2.75	(2.49)
Neck pain (NDI) (0–100)^b^	24.18	(8.22)	22.80	(6.20)	24.94	(12.87)	19.07	(12.50)
Concerns of falling (FES-I) (16–64)^b^	29.00	(5.71)	26.70	(6.29)	26.00	(5.61)	24.42	(5.21)
SF12 PCS (0–100)^c^	36.20	(8.45)	40.18	(10.98)	42.12	(6.91)	43.96	(10.01)
SF12 MCS (0–100)^c^	49.20	(10.79)	49.98	(8.71)	47.76	(9.75)	52.90	(9.45)
Mood								
Depression (DASS) (0–21)^b^	8.55	(4.99)	7.20	(6.20)	5.38	(4.03)	3.50	(4.52)
Anxiety (DASS) (0–21)^b^	8.73	(6.28)	6.20	(6.43)	6.00	(2.58)	4.50	(2.97)
Stress (DASS) (0–21)^b^	10.73	(5.61)	7.60	(5.48)	9.08	(6.09)	8.33	(7.02)
Physical function								
Functional reach (cm)^d^	32.41	(5.90)	30.60	(10.30)	29.93	(11.75)	31.25	(8.37)
Step test sum (n)^d^	27.09	(6.16)	26.10	(7.08)	25.46	(7.09)	26.08	(8.45)
Four square step test (seconds)^e^	11.20	(2.51)	14.18	(8.24)	11.92	(2.95)	11.22	(3.18)
Timed up & go (seconds)^e^	12.09	(2.87)	12.36	(4.11)	12.18	(2.70)	11.87	(3.67)
Correctly identified which group they were allocated to (n)			5	(50%)			8	(67%)
Treatment satisfaction (1–5)^f^			3.60	(0.70)			3.58	(1.00)

Values displayed as mean(standard deviation) or number(percentage)

*M* mean, *SD* standard deviation, *MoCA* Montreal cognitive assessment, *NDI* neck disability index, *DHI* dizziness handicap inventory, *PCS* physical health composite score, *MCS* mental health composite score, *DASS* depression anxiety and stress scale.

^a^a lower score indicates reduced cognitive function

^b^a higher score indicates greater symptoms

^c^a higher score indicated greater quality of life

^d^a higher score indicates greater physical function

^e^a faster time indicates greater physical function

^f^1-I feel much worse 2-I feel worse 3-I feel the same 4-I feel better 5-I feel much better

**Table 3:** Proportion of improvement in primary clinical outcomes of NDI and DHI in each group

Sham group (n = 10)				Chiropractic group (n = 12)		
% Improvement	MCID*	30%	50%	MCID*	30%	50%
NDI	30%	10%	0%	58%	33%	25%
% Improvement	MCID*	30%	50%	MCID*	30%	50%
DHI	50%	40%	20%	67%	33%	25%

*Minimal Clinically Important Difference (MCID) for NDI is 19% [48] and for DHI is 18% [56]

### Sample size calculation

The sample size for a fully-powered trial (derived from data in this feasibility trial with an effect size of *d* = 0.38), using the DHI as the primary outcome measure, would require a group size of 150 (i.e. 75 per group). Alternatively, using NDI as the primary outcome measure (with an effect size of *d* = 0.46), would require a group size of 222 (i.e. 111 per group). These calculations exclude provision for XX%? drop-outs.

## Discussion

A fully-powered trial based on the current study would not be feasible in our setting using the current protocol. However, a trial may be feasible with modifications to the study location and recruitment strategies. Recruitment of this study achieved sufficient numbers to calculate sample sizes for potential larger trials. Blinding was acceptable in both groups.

### Recruitment

We aimed for a sample size of 40 participants. However, recruitment did not reach this pre-defined arbitrary number, even with a six-month extension to the recruitment period. If conducted in the same setting, to reach the estimated sample size of 150 participants (using DHI as the primary outcome measure) would take more than four years, assuming a similar recruitment rate. This study found that using online recruitment methods could be useful in targeting older Australians over a wider geographic area. On the other hand, newspaper advertisements, while more expensive, captured a local population. Considering the relatively high proportion (12%) of participants who could not make the travel commitment to our single location, for future studies we recommended having multiple sites with sufficient geographical spread to increase recruitment and retention or performing the study in a facility with a high concentration of elderly people with neck pain and dizziness such as a specialty clinic.

Older adults experiencing neck pain and dizziness often have co-morbidities. However, chiropractic intervention is unlikely to impact dizziness due to known vestibular or neurological origin, dizziness as a result of postural hypotension, or poly-pharmacy. Therefore, we recommend that the same exclusion of people with vestibular or neurological causes of dizziness be used for the larger study. Excluding these participants based on self-reported previous diagnoses, however, may not capture these individuals accurately. We recommend basing a future, larger trial in a rehabilitation setting such as a dizziness/falls clinic which provides access to clinical expertise and equipment to rule out vestibular, neurological, hypotensive and pharmacological causes of dizziness. Alternatively, the larger trial can be based on an effective referral system from a network of general medical practitioners. Exclusion of people based on cognitive-function testing has been shown to reduce the generalizability of findings [46], particularly in older people with pain [47]. However, the validity of self-reported measures of pain and function depends on intact memory and executive function. Participants in this trial who were excluded based on cognitive function were significantly disappointed to the extent that one of them lodged a complaint to the ethics committee. Future studies should consider how participants with potential impairments in cognitive function can be included, using outcome measures that are still able to capture self-reported pain and function. Alternatively, if a threshold of cognitive function is used as an exclusion criteria in future studies, procedures need be in place to direct excluded participants to providers of therapeutics for neck pain and dizziness to avoid disappointment.

### Compliance with outcome assessment

The assessment regime was not too onerous for the participants and was completed in a timely manner. However, several participants missed individual questions on the questionnaires, and had to be prompted by the investigator to fill these in. It was necessary to have an investigator review completed questionnaires to check that all questions had been completed at the end of the assessment session.

### Compliance with the intervention

The drop-out rate for participants was acceptable, with less than 15% for each group. However, this was for a relatively short intervention schedule of 4 visits over 4 weeks. It cannot be determined from this study if a longer, more intensive or less intensive schedule would have good compliance.

### Location

Conducting the trial at a university campus meant that some participants became lost trying to find the building location. Future studies should be conducted in an easy to find location with convenient car parking facilities, and ideally with a choice of several sites to capture participants who cannot travel long distances.

### Interventions and blinding

The protocol of using the Activator II™ instrument (set on zero) as a sham-chiropractic intervention appeared to achieve sufficient blinding in participants. This tool appears to be a useful blinding tool for future similar studies, particularly ones in which the experimental intervention consists of Activator II™-instrument delivered manipulation.

### Costs

The costs and time to recruit sufficient numbers may be a challenge for a larger fully-powered RCT. Use of a network of chiropractic intervention sites may increase feasibility of recruitment. The cost of AUD$2415 per participant may prove prohibitive if only small grant funding is available. To reduce this expenditure, the larger study could be based in a dizziness/falls clinic of a general or rehabilitation hospital. The use of a specialised or hospital recruitment setting would necessitate modification of this protocol, and our results may not be reflective of the participants recruited in such settings.

### Harms

Fifteen out of 23 participants did not report any harms. Mild harms such as transient increases in neck pain or headache are common following chiropractic intervention [28]. However, participants in the sham group also reported these harms, so these may be related to natural and non-specific effects [44].

### Strengths and limitations

Trials of non-pharmacological interventions for pain and dizziness in older people are scarce. This trial provides useful information in the Australian context on recruiting older people, and blinding for spinal manipulation, both of which are challenging. This is important information for future research. Furthermore, this was a feasibility study for determining effectiveness rather than efficacy. This necessitated that the intervention given reflected a ‘real-world’ combination of intervention strategies that Australian chiropractors would provide. Effectiveness studies by nature are not mechanistic and cannot identify the ‘active ingredient’ in the intervention package. But they do have higher external validity in their relevance and applicability to actual practice. In this sense, this was a trial comparing usual chiropractic care with sham chiropractic care. The intervention combination used here reflects the practice approach of a majority of Australian chiropractors (unpublished data), and follows contemporary practice guidelines for the treatment of the elderly [38]. However, it does not reflect every chiropractor’s practice style, particularly in its exclusion of manual manipulation of the neck. This limits the relevance of this study to trials of manual neck manipulation, as the biomechanics of manual manipulative thrusts are likely to be different from those delivered by an Activator instrument.

This trial was limited by the short-term follow-up, and no conclusions can be drawn about compliance with longer follow-up times. While the results of this trial advocate for conducting a fully-powered RCT at multiple locations, it did not test the feasibility of a protocol to ensure consistent recruitment and data collection across several sites. These issues should be investigated before such large-scale multi-centre studies are attempted. Another limitation of this study is that the participants were excluded based on self-reported previous diagnoses of dizziness, and were not uniformly screened by specialist medical staff to exclude other causes of dizziness. This may have made the cohort of participants somewhat heterogeneous. However, this heterogeneity reflects private practice that takes place within the primary care setting. Furthermore, this study is limited by including participants with very low intensities of dizziness and neck pain. There was no threshold for severity or intensity of dizziness or neck pain for inclusion. Setting of minimum DHI and NDI scores as inclusion criteria for future studies is recommended, although this would lead to a lower proportion of interested participants being eligible.

## Conclusions

A large trial in an Australian university setting using the current protocol is not likely to be feasible primarily for financial and recruitment reasons. However, a fully-powered clinical trial may be feasible at an appropriate hospital or rehabilitation setting, which would require sample size of 150 (75 per group) or 222 (111 per group) using DHI or NDI as the primary outcome measure respectively. Activator II™-instrument-assisted sham intervention provided acceptable blinding. The number and nature of the outcome measures used was not too onerous for the participants.

## Abbreviations

DASS: Depression Anxiety Stress Scales
DHI: Dizziness Handicap Inventory
FESI: Falls Efficacy Scale International
NDI: Neck Disability Index
NRS: Numerical Rating Scale
RCT: Randomised Controlled Trial
SMT: Spinal Manipulative Therapy
